# Supplementary material for: Content-rich biological network constructed by mining PubMed abstracts
Source: BMC Bioinformatics. 2004 Oct 8;5:147. doi: 10.1186/1471-2105-5-147 (PMC528731; doi:10.1186/1471-2105-5-147)
Supplement: Additional File 2 — The original results of the above study (non-essential files are deleted to keep the file size under the limit set by BMC bioinformatics). [file 1471-2105-5-147-S2.bz2 › chilibotAdditionalFile2/dip30/713ID8175697E18050/html/SECA_SECB.html]

 


 **SECA** and **SECB** 
  
Found 76 abstracts in PubMed, retrieved 30.  
 

 What does Google say? 
 PDF only 
| .edu only 

---

**Interactive relationship** (e.g. stimulation, inhibition, etc)

**Stimulatory relationship**- Evidence that  **SecB**  enhances the activity of  **SecA** .  Ref: 11297435 Biochemistry, 2001
- **SecB**  is also shown to directly enhance the ATPase activity of  **SecA**  in a concentration dependent and saturable manner.  Ref: 11297435 Biochemistry, 2001
- Biochemical studies suggest that  **SecB**  facilitates precursor targeting by binding to the  **SecA**  protein, a component of the membrane embedded translocation apparatus.  Ref: 10322000 J Bacteriol, 1999
- Ffh, as well as  **SecB**  in Escherichia coli, enhances the binding of  **SecA**  to presecretory proteins in B. subtilis cells.  Ref: 9880811 J Biochem Tokyo , 1999
- The results suggest that  **SecB**  directly enhances the activity of  **SecA**  and provide a biochemical basis for the enhanced transport efficiency of preproteins in the presence of  **SecB**  in vivo.  Ref: 11297435 Biochemistry, 2001
- Treatment of  **SecA**  with a zinc chelator resulted in a loss of the stimulatory effect of  **SecB**  on the  **SecA**  translocation ATPase activity, while the activity could be restored by the addition of ZnCl2.  Ref: 10213615 Biochemistry, 1999
- The equilibrium dialysis experiments and kinetic data support the observation that the  **SecB**  enhanced  **SecA**  ATPase activity is the result of an increased rate of ATP hydrolysis rather than an increase in the affinity of ATP for  **SecA** .  Ref: 11955083 Biochemistry, 2002
**Neutral relationship**- The molecular chaperone  **SecB**  targets preproteins to  **SecA**  at the translocation sites in the cytoplasmic membrane of Escherichia coli.  Ref: 10213615 Biochemistry, 1999
- **SecA**  recognizes  **SecB**  via its carboxyl terminal 22 aminoacyl residues, a highly conserved domain that contains 3 cysteines and 1 histidine residue that could potentially be involved in the coordination of a metal ion.  Ref: 10213615 Biochemistry, 1999
- The variant forms of  **SecB**  were all capable of interacting with  **SecA**  in solution to form complexes with stability similar to that of complexes between  **SecA**  and wild type  **SecB** .  Ref: 10807917 J Biol Chem, 2000
- Therefore, deletion of the mobile region of  **SecB**  may alter the interactions of  **SecB**  with  **SecA** .  Ref: 10544036 Biochem Biophys Res Commun, 1999
- Interaction of  **SecB**  with the  **SecB**  binding domain of  **SecA**  is disrupted by chelators of divalent cations, and could be restored by the addition of Cu2 or Zn2.  Ref: 10213615 Biochemistry, 1999
- In Escherichia coli, the formation of  **SecA**   **SecB**  complexes has a direct effect on  **SecA**  ATPase activity.  Ref: 11955083 Biochemistry, 2002
- We present evidence that at least two regions of  **SecA**  are involved in the formation of active complexes with  **SecB** .  Ref: 10807917 J Biol Chem, 2000
- **SecB**  uses its entire molecular surface for these two functions, but for preprotein release and its own membrane release,  **SecB**  relies on the catalytic activity of  **SecA** .  Ref: 11336818 Trends Microbiol, 2001
- In this latter role  **SecB**  demonstrates specific binding to a unique partner,  **SecA** .  Ref: 12475171 Cell Mol Life Sci, 2002
- These findings lend additional support to the proposed pathway for precursor targeting in which  **SecB**  promotes targeting to the translocation apparatus by binding to the  **SecA**  protein.  Ref: 10322000 J Bacteriol, 1999
- The crystal structure of the Haemophilus influenzae  **SecB**  provides new insights into how  **SecB**  simultaneously recognizes its two ligands unfolded preproteins and  **SecA** , the ATPase subunit of the translocase.  Ref: 11336818 Trends Microbiol, 2001
- It is concluded that the  **SecB**  binding domain of  **SecA**  is stabilized by a zinc ion that promotes the functional binding of  **SecB**  to  **SecA** .  Ref: 10213615 Biochemistry, 1999
- To gain more insight into the functional interaction of  **SecB**  and  **SecA** , in vivo, mutations in the  **secA**  locus that compensate for the export defect caused by the  **secB**  missense mutation secBL75Q were isolated.  Ref: 10322000 J Bacteriol, 1999
- The Escherichia coli secretion dedicated chaperone  **SecB**  targets a subset of proteins to the translocase by interacting with the carboxyl C terminus of  **SecA** .  Ref: 11021932 Microbiology, 2000
- It appears that  **SecB**  may function as an intermolecular regulator of ATP hydrolysis by promoting the ATP bound state of  **SecA** .  Ref: 11955083 Biochemistry, 2002
- During localization to the periplasmic space or to the outer membrane of Escherichia coli some proteins are dependent on binding to the cytosolic chaperone  **SecB** , which in turn is targeted to the membrane by specific interaction with  **SecA** , a peripheral component of the translocase.  Ref: 10807917 J Biol Chem, 2000
- Using a chemical cross linking approach, we find that the formation of  **SecA**   **SecB**  complexes is accompanied by a decrease in the level of cross linking of  **SecA**  dimers, suggesting that  **SecB**  induces a conformational change in  **SecA** .  Ref: 11297435 Biochemistry, 2001
- In contrast,  **SecB**  is utilized by only a subset of preproteins to prevent their premature folding and chaperone them to membrane bound  **SecA** .  Ref: 11297435 Biochemistry, 2001

**Non-interactive relationship** (e.g. studied together, co-existance, homology, etc.)

- An artificial transmembrane segment directs  **SecA** ,  **SecB** , and electrochemical potential dependent translocation of a long amino terminal tail.  Ref: 10037778 J Biol Chem, 1999
- Overproduction of  **SecA**  suppresses the export defect caused by a mutation in the gene encoding the Escherichia coli export chaperone  **secB** .  Ref: 10322000 J Bacteriol, 1999
- The integration of two membrane proteins into inside out plasma membrane vesicles of E. coli required all three SRP components and could not be driven by  **SecA** ,  **SecB** , and DeltamicroH.  Ref: 10397756 Mol Biol Cell, 1999
- These data suggest that SRP FtsY and  **SecB**   **SecA**  constitute distinct targeting routes.  Ref: 10514469 J Biol Chem, 1999
- ... secretory proteins are directed into the  **SecA**   **SecB**  mediated post translational targeting pathway by means of their preferential recognition by trigger factor.  Ref: 10619852 EMBO J, 2000
- In Escherichia coli, the  **SecB**   **SecA**  branch of the Sec pathway and the twin arginine translocation Tat pathway represent two alternative possibilities for posttranslational translocation of proteins across the cytoplasmic membrane.  Ref: 12700260 J Bacteriol, 2003
- Recent evidence suggests that in Escherichia coli,  **SecA**   **SecB**  and signal recognition particle SRP are constituents of two different pathways targeting secretory and inner membrane proteins to the SecYEG translocon of the plasma membrane.  Ref: 10931878 J Cell Biol, 2000
- Export after  **SecA**  depletion was observed in cells that lack  **SecB** , the primary targeting factor for OMPs, but was abolished by partial inactivation of DnaK.  Ref: 12403776 J Biol Chem, 2002
- Besides  **SecA**  and  **SecB** , Escherichia coli cells possess a signal recognition particle SRP to target exported proteins to the SecY translocon.  Ref: 10619852 EMBO J, 2000
- Discrimination between SRP and  **SecA**   **SecB**  dependent substrates involves selective recognition of nascent chains by SRP and trigger factor.  Ref: 10619852 EMBO J, 2000
- The characterization of a spontaneous TorA signal peptide mutant TorA\*, in which the two arginine residues in the c region had been replaced by one leucine residue, showed that the TorA\* MalE mutant precursor had acquired the ability for efficiently using the  **SecB**   **SecA**  pathway.  Ref: 12700260 J Bacteriol, 2003
- **SecB**  modulates the nucleotide bound state of  **SecA**  and stimulates ATPase activity.  Ref: 11955083 Biochemistry, 2002
- Furthermore, functional signal peptides, but not dysfunctional ones, promote the formation of  **SecA**   **SecB**  complexes.  Ref: 11297435 Biochemistry, 2001
- The proteolysis data indicate that  **SecB**  and the nonhydrolyzable ATP analogue AMP P C P induce similar conformational changes in  **SecA**  which result in a more open or extended structure that is suggestive of the ATP bound form.  Ref: 11955083 Biochemistry, 2002
- This region of  **SecA**  is highly conserved in Eubacteria, but despite its presence in the Bacillus subtilis  **SecA** , the B. subtilis genome does not appear to contain a gene for a clear homologue of  **SecB** .  Ref: 11021932 Microbiology, 2000
- Complexes between protein export chaperone  **SecB**  and  **SecA** .  Ref: 10807917 J Biol Chem, 2000
- In vitro studies with purified components reveal signal recognition particle SRP and  **SecA**   **SecB**  as constituents of two independent protein targeting pathways of Escherichia coli.  Ref: 10397756 Mol Biol Cell, 1999
- The incorporation of sequence motifs in the mature region, which confer  **SecB**  dependence in vivo, had no impact on  **SecA**  activation in vitro.  Ref: 11969418 Biochemistry, 2002
- Using purified components and synthetic signal peptides, we have studied the interaction of  **SecB**  with  **SecA**  and with  **SecA**  signal peptide complexes in vitro.  Ref: 11297435 Biochemistry, 2001
- This was achieved in a novel cell free system from E. coli which, by extensive subfractionation, was simultaneously rendered deficient in  **SecA**   **SecB**  and the signal recognition particle SRP components, Ffh P4.  Ref: 10397756 Mol Biol Cell, 1999
- The inhibition of  **SecA**  ATPase activity by sodium azide in the presence of IMVs and a functional signal peptide further indicates that  **SecB**  promotes the ATP bound form of  **SecA** .  Ref: 11955083 Biochemistry, 2002
- These results indicate that SRP dependent integration and  **SecA**   **SecB**  mediated translocation do not only represent two independent protein delivery systems, but also remain mechanistically distinct processes even at the level of the membrane where they engage different domains of SecY and different components of the translocon.  Ref: 10931878 J Cell Biol, 2000
- In concert with  **SecB** ,  **SecA**  targets the precursors to SecY and energizes their translocation by its ATPase activity.  Ref: 11051763 Prog Nucleic Acid Res Mol Biol, 2001
- In Escherichia coli, three different types of proteins engage the SecY translocon of the inner bacterial membrane for translocation or insertion 1 polytopic membrane proteins which prior to their insertion into the membrane are targeted to the translocon using the bacterial signal recognition particle SRP and its receptor, 2 secretory proteins which are targeted to and translocated across the SecY translocon in a  **SecA**  and  **SecB**  dependent reaction and 3 membrane proteins with large periplasmic domains, requiring SRP for targeting and  **SecA**  for the translocation of the periplasmic moiety.  Ref: 12682042 J Biol Chem< MedlineTA>J Biol Chem, 2001
- **SecA**  dependent binding of E. coli  **SecB**  to membrane vesicles was observed when E. coli  **SecA**  was used, but not with the S. lividans  **SecA** , suggesting that this interaction may be specific for the Gram negative bacteria.  Ref: 9826195 Eur J Biochem, 1998
- Zinc stabilizes the  **SecB**  binding site of  **SecA** .  Ref: 10213615 Biochemistry, 1999
- To determine the biological consequence of this finding, the influence of  **SecB**  on the signal peptide stimulated  **SecA**  lipid ATPase was studied using synthetic peptides of varying hydrophobicity.  Ref: 11297435 Biochemistry, 2001
- In contrast, here we describe alkaline phosphatase mutants containing artificial transmembrane segments that demonstrate that translocation of a long N tail across the membrane is dependent upon  **SecA** ,  **SecB** , and the electrochemical potential in the absence of a signal peptide.  Ref: 10037778 J Biol Chem, 1999
- Discrimination between  **SecA**   **SecB**  and SRP dependent targeting involves the specificity of SRP for hydrophobic signal anchor sequences and the exclusion of SRP from nascent chains of translocated proteins by trigger factor, a ribosome associated chaperone.  Ref: 11051763 Prog Nucleic Acid Res Mol Biol, 2001
- Furthermore, they suggest that SRP and  **SecA**   **SecB**  have different substrate specificities resulting in two separate targeting mechanisms for membrane and secretory proteins in E. coli.  Ref: 10397756 Mol Biol Cell, 1999
